# Norepinephrine triggers an immediate-early regulatory network response in primary human white adipocytes

**DOI:** 10.1186/s12864-018-5173-0

**Published:** 2018-11-03

**Authors:** Juan Carlos Higareda-Almaraz, Michael Karbiener, Maude Giroud, Florian M. Pauler, Teresa Gerhalter, Stephan Herzig, Marcel Scheideler

**Affiliations:** 1 Institute for Diabetes and Cancer (IDC), Helmholtz Zentrum München, German Research Center for Environmental Health, Neuherberg, Germany; 2 Joint Heidelberg-IDC Translational Diabetes Program, Heidelberg University Hospital, Heidelberg, Germany; 3 Molecular Metabolic Control, Medical Faculty, Technical University, Munich, Germany; 4 German Center for Diabetes Research (DZD), Neuherberg, Germany; 5 Department of Phoniatrics, ENT University Hospital, Medical University of Graz, Graz, Austria; 6 CeMM, Research Center for Molecular Medicine of the Austrian Academy of Sciences, Vienna, Austria; 7 Present Address: Institute of Science and Technology (IST) Austria, Klosterneuburg, Austria; 8 NMR laboratory, Institute of Myology, Hopital Universitaire Pitie Salpetriere, Paris, France

**Keywords:** Norepinephrine stimulation, White adipocyte, Early cell fate, Network biology, Immediate-early gene, Transcriptional regulatory network

## Abstract

**Background:**

Norepinephrine (NE) signaling has a key role in white adipose tissue (WAT) functions, including lipolysis, free fatty acid liberation and, under certain conditions, conversion of white into brite (brown-in-white) adipocytes. However, acute effects of NE stimulation have not been described at the transcriptional network level.

**Results:**

We used RNA-seq to uncover a broad transcriptional response. The inference of protein-protein and protein-DNA interaction networks allowed us to identify a set of immediate-early genes (IEGs) with high betweenness, validating our approach and suggesting a hierarchical control of transcriptional regulation. In addition, we identified a transcriptional regulatory network with IEGs as master regulators, including HSF1 and NFIL3 as novel NE-induced IEG candidates. Moreover, a functional enrichment analysis and gene clustering into functional modules suggest a crosstalk between metabolic, signaling, and immune responses.

**Conclusions:**

Altogether, our network biology approach explores for the first time the immediate-early systems level response of human adipocytes to acute sympathetic activation, thereby providing a first network basis of early cell fate programs and crosstalks between metabolic and transcriptional networks required for proper WAT function.

**Electronic supplementary material:**

The online version of this article (10.1186/s12864-018-5173-0) contains supplementary material, which is available to authorized users.

## Background

White adipose tissue (WAT) is a multifunctional organ that governs energy storage, endocrine functions and signaling for maintaining energy homeostasis in the body [1]. WAT also has the capacity to expand in response to caloric intake, hormones, and in the aging process [2]. As a counterpart to WAT, brown adipose tissue (BAT), which is located predominantly in the interscapular area in human adults, maintains thermoregulation of the body during acute or prolonged cold exposure. It is equipped with a high density of mitochondria and multilocular lipid droplets [3]. The thermogenic function of BAT relies mainly on UCP1, a mitochondrial protein that uncouples oxidative phosphorylation from ATP synthesis, leading to energy dissipation [4]. Recently, the remodeling of white adipocytes to brown-like fat cells (brite) has been reported, with characteristics such as UCP1 expression and thermogenesis that resemble BAT [5]. The brite adipocyte upraise has been observed upon exposure to cold or in response to different stimuli, such as chronic exposition to rosiglitazone (Rosi), a PPARγ agonist [6], Celastrol, a plant-derived triterpene [7], and norephineprhine (NE) [8].

NE is an integral part of the sympathetic nervous system and mediates essential physiologic responses, including increased heart rate and blood pressure, mobilization of energy stores and control of core body temperature [9]. NE exerts its effects by binding to adrenergic receptors α and β, linked to G_S_ proteins [10], which in turn are linked to an adenylate cyclase. NE binding thus causes a rise in the intracellular concentration of cyclic AMP (cAMP). Downstream effectors of cAMP include cAMP dependent protein kinase (PKA), which mediates most of the so far known intracellular events following NE binding [11]. In WAT, it is well known that adrenergic stimuli trigger lipolysis and mobilization of free fatty acids [12]. In BAT, NE-activated PKA can also phosphorylate p38 MAPK, which activates the transcription of UCP1 by phosphorylating the PPARγ coregulator 1α (PGC1α) codified by the gene PPARGC1A, and the transcription factor ATF [13–15].

It is reasonable to conclude that, in response to cold, signaling by NE can induce well-differentiated cellular programs in different adipose tissues. In BAT, NE triggers a thermogenic program dependent on a signaling cascade that leads to elevated UCP1 expression and activation of UCP1 by lipolysis [16]. In WAT two programs are carried out: lipolysis leads to fat mobilization, immediately executed and dependent almost exclusively on a phosphorylation cascade of existing proteins [17]. In addition, conversion of WAT to the brown-like phenotype implies a major transcriptional shift, caused by a profound remodeling of the superenhancers responsible for the maintenance of adipogenesis [18], the down-regulation of pro-adipogenic transcription factors (TFs) such as PPARγ, and the initiation of the transcriptional program that triggers thermogenesis [19]. However, while long-term NE-triggered phenotypical and physiological effects are known, immediate-early transcriptional responses to NE are still obscure.

Cell-extrinsic signals can activate a specific set of genes, called immediate-early genes (IEGs), which are transcribed within minutes after stimulation, are expressed in waves without the need for de novo protein synthesis [20, 21], and are transcription factors (TFs) that can control target gene expression. IEGs have emerged to respond to a variety of extrinsic stimuli in multiple cell types, indicating a common response mechanism probably comprising several hundred genes [22]. Nevertheless, little is known about IEGs that are triggered by NE in adipocytes.

The coordination of regulatory mechanisms becomes critical for an accurate gene expression pattern in biological processes, and this transcriptional regulation is structured into a hierarchical organization with regulators at different levels exhibiting unique characteristics [23]. Standard transcriptome analyses typically uncover expression changes for hundreds or thousands of genes. However, without additional system level approaches, they are not able to reveal the complex mechanisms behind changes in gene expression. Simplistic approaches have failed so far to characterize the complex cellular response defined by the collective contribution of regulatory and signaling pathways [24, 25]. As no single TF is sufficient to drive a complex regulatory process alone, changes in cellular states are determined by complex networks, involving both positive and negative regulatory interactions with a substantial number of TFs [26]. In the context of adipocytes, the early complex cellular response to NE and its underlying transcriptional regulatory network still awaits to be studied.

In order to get insight into this complexity, it is necessary to study molecules in a network context, including protein-protein interactions (PPI) and metabolic, signaling and transcriptional regulatory networks (TRN) [27]. The sum of all these interactions, the cellular network, allows to elucidate and visualize complex interactions and their information flow, where molecules are represented as nodes and their interaction as edges [28]. The analysis of structural network components and their behavior using topology offers a quantifiable measurement that provides insight into biological functions of cellular networks. The analysis of betweenness centrality, for example, allows the identification of highly interconnected nodes known as “bottlenecks”, which may point toward their essentiality [29, 30]. Therefore, the complex relationships between components can only be elucidated from a network perspective [31]. Moreover, integration of functional clustering and analysis of network topology paves the way to disclose new associations among genes cooperating in undisclosed, not yet annotated biological processes [32, 33].

Here, using these tools and concepts, we investigated the early transcriptional response of human white adipocytes to 3 h of NE stimulation. First, we found a broad transcriptional response with more than 2,100 differentially expressed genes assigned to a wide range of pathways. Second, we inferred the transcriptional regulatory network of these NE-responsive genes, which indicated that the known and novel immediate-early genes might serve as master and local regulators. Third, we performed a functional enrichment analysis that suggested the participation of several genes as a “bridge” between functional clusters. These transcriptional and post-transcriptional regulators and their target genes provide a novel insight into the circuitry and functional principles of the acute response to NE in human white adipocytes.

## Results

### NE stimulation triggers an acute and broad transcriptional response in primary human white adipocytes

**Fig. 1:**
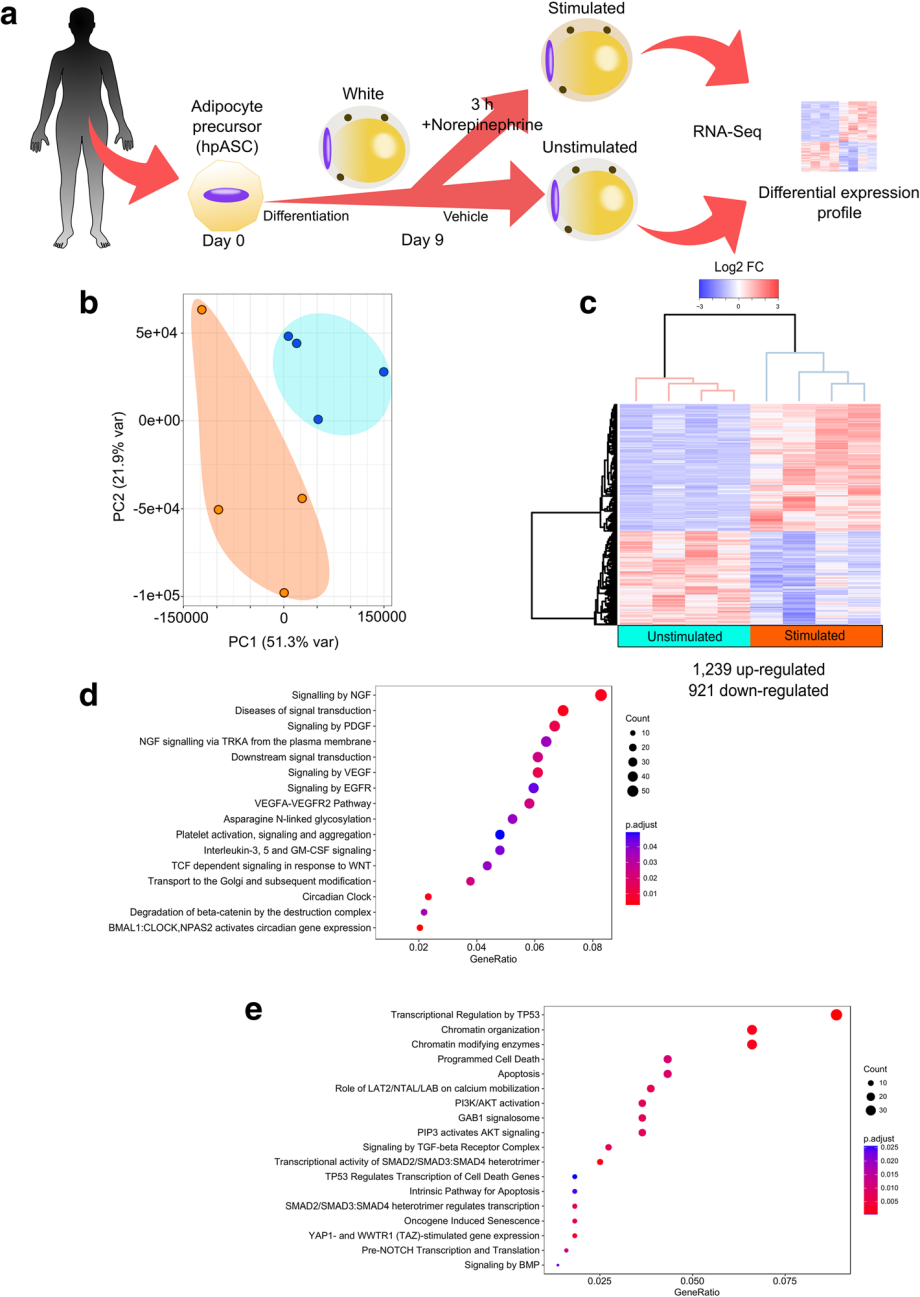
NE stimulation triggers an acute and broad transcriptional response in human adipocytes. **a** Workflow diagram for sample preparation. Adipocyte precursor cells were isolated from donors (*n* = 4), differentiated and treated with NE or vehicle for 3 h, harvested and subsequently analyzed by RNA-seq (The female human silhouette was modified from a clipart with a public domain license creative commons CC0). **b** Principal component analysis (PCA) and unsupervised hierarchical clustering (**c**) showed clustering between stimulated (orange) and unstimulated (blue) adipocytes in two distinct groups, suggesting that NE stimulation causes deep changes in the transcriptome. Columns are biological replicates and rows represent differentially expressed genes. **d** Reactome pathway enrichment for up- and **e** down-regulated DEGs after acute NE stimulation

In order to investigate the early response of primary human adipocytes to NE stimulation, we obtained human primary adipose-derived stem cells (hpASCs) from four female individuals. These hpASCs were exposed to an adipogenic cocktail, permitting the development of terminally differentiated white adipocytes within 9 days. These mature adipocytes were then treated with NE (i.e. stimulated) or vehicle (i.e. unstimulated), harvested after 3 h, and used to perform RNA-seq analysis (Fig. 1a).

We performed a principal component analysis (PCA) of the RNA-seq data, which revealed that the expression signatures from the stimulated adipocytes separate from the unstimulated ones in each biological replicate (Fig. 1b). Differential gene expression analysis of stimulated compared to unstimulated adipocytes showed 2,160 differentially expressed genes (DEGs) that were significantly up-regulated (1,239) or down-regulated (921) (Fig. 1c). Among the up-regulated genes we found CREM [34] (1.5 log_2_FC) and PPARGC1A [35] (2.5 log_2_FC), both known to be up-regulated in response to NE, thereby validating our experimental approach.

In order to ascertain which pathways were acutely stimulated in white adipocytes upon NE treatment, we performed a pathway enrichment analysis using the Reactome database. Up-regulated genes were enriched for circadian cycle and gene-related signaling pathways (Fig. 1d), primarily nerve growth factor (NGF), platelet-derived growth factor (PDGF), VEGF, and WNT/Beta-catenin. On the other hand, enriched pathways for the down-regulated genes comprise p53 downstream targets, chromatin organization, apoptosis regulators, GAB1 signalosome super pathway (which includes PI3K/AKT/LAT2/LAB pathways), signaling by TGFβ receptor complex, and targets of SMAD heterodimers (Fig. 1e). The large number of alterations in gene expression suggests an acute transcriptional response, while the finding of many different modulated pathways postulates a broad transcriptional response to NE in primary human white adipocytes.

### Acute NE responsive network has immediate-early genes as nodes with high betweenness

**Fig. 2:**
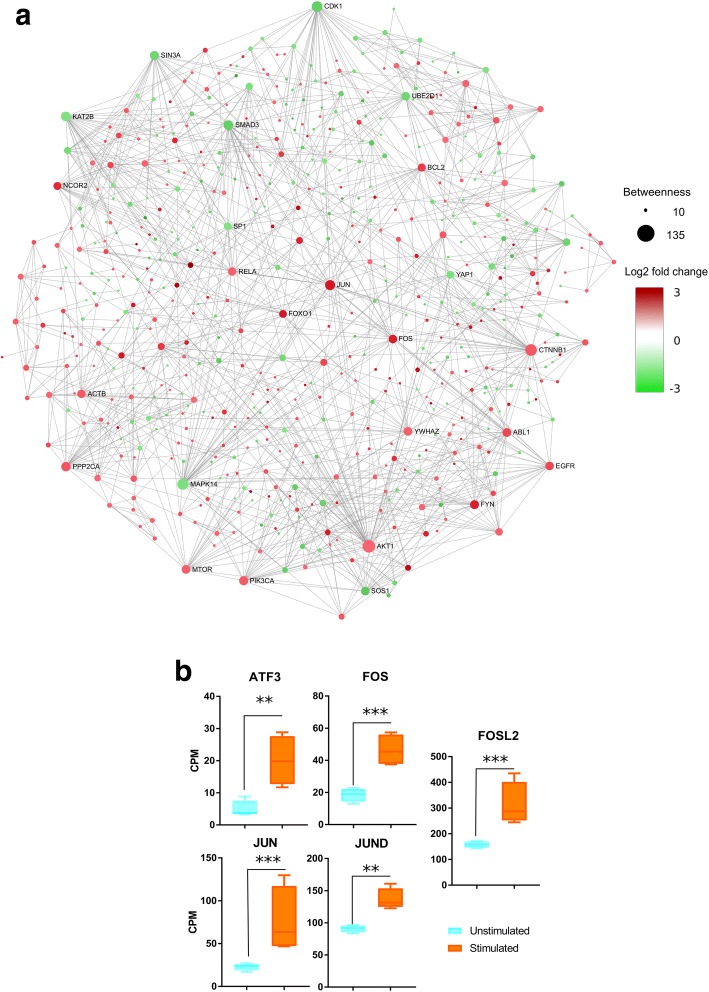
Acute NE responsive network has immediate-early genes as nodes with high betweenness. **a** The interaction network reconstructed from DEGs shows nodes with high betweenness. Edges represent experimentally verified protein-protein or protein-DNA interactions. Up-regulated DEGs are in red and down-regulated DEGs in green. Size node is related to betweenness value. **b** Known immediate-early genes were found to be up-regulated after NE stimulation. Expression values are shown in log_2_ counts per million (CPM). Expression differences between conditions were analyzed with a Kruskall-Wallis Test, *P* < 0.0001, Dunn’s Multiple Comparison Test *P* < 0.05; ET Test, P < 0.05 (*** *p* < 0.0001, ** *p* < 0.001)

In order to gain insight into the molecular network triggered acutely by NE stimulation, we first constructed the experimentally verified interactions within up- and down-regulated DEGs by inferring their PPI and Protein-DNA networks. Second, we explored the connection between network structure and global network topology. The betweenness of all the nodes in the network was calculated using the Cytoscape [36] plug-in cytoHubba [37]. The nodes with the highest betweenness values in the network of up-regulated genes were JUN, FOS, NCOR2, FOXO1, CEBPB, TLE1, CREM, ATF3, NR4A1, EPHB2, HIPK2 and RHOB (Fig. 2a, Additional file 1: Table S1). JUN and FOS are described as IEGs [38], known to be transcribed in other models, during adipogenesis [39] and upon adrenergic stimulation [40]. However, the participation of these TFs in acute NE response in mature adipocytes was not yet known until now. In the network of down-regulated genes, the top nodes – ranked by betweenness – were SMAD3, FYN, RUNX2 and SOS1 (Fig. 2a, Additional file 1: Table S1). SMAD3 [41] and RUNX2 [42] are known to participate in adipocyte differentiation from precursors but not in early NE stimulation.

Since JUN and other IEGs were up-regulated genes with higher betweenness within the network, we measured expression levels of JUN and other known IEGs such as JUND, ATF3, FOS and FOSL2 in detail. These genes were up-regulated in adipocytes stimulated with NE, demonstrating that NE stimulation activates IEG transcription (Fig. 2b).

All together, these results shows that several genes with high betweenness are IEGs. These findings suggest a transcriptional regulatory network organized by highly connected genes, identified as IEGs in the very early response to NE stimulation.

### Transcriptional regulatory network analysis reveals immediate-early genes responsive to NE stimulation

Since we observed altered transcription of known IEGs, we were interested in getting insight into the emergent properties of the acute NE-treated adipocyte regulation by looking into the TRN. Therefore, we classified the TFs according to hierarchy as global (“master”) regulators (MRs) or mid-level (“local”) regulators (LRs). MRs are genes at the top of the gene regulatory hierarchy that regulate multiple downstream genes either directly or through a cascade of gene expression changes, and have the ability to re-direct the fate of cells [43]. LRs are defined as those TFs that are regulated by MRs, and are mostly dedicated to regulate a specific set of downstream targets [44]. In order to identify the regulatory hierarchy in our TRN, a TF motif activity prediction was performed on the networks of our significantly up- and down-regulated genes using iRegulon [45]. TFs with high, normalized enrichment scores (NES ≥ 3) were identified in each network, corresponding to an estimated false discovery rate of less than 0.01, which we used as threshold for our dataset. In the transcriptional regulatory network of up-regulated TFs we found a total of 147 overrepresented TFs, 60 of them with differentially expressed targets, comprising 10 MRs and 50 LRs (Additional file 1: Table S2). In the regulatory network of down-regulated TFs, we found a total of 143 TFs, 81 of them with differentially expressed targets, comprising four MRs and 77 LRs (Additional file 1: Table S3).

**Fig. 3:**
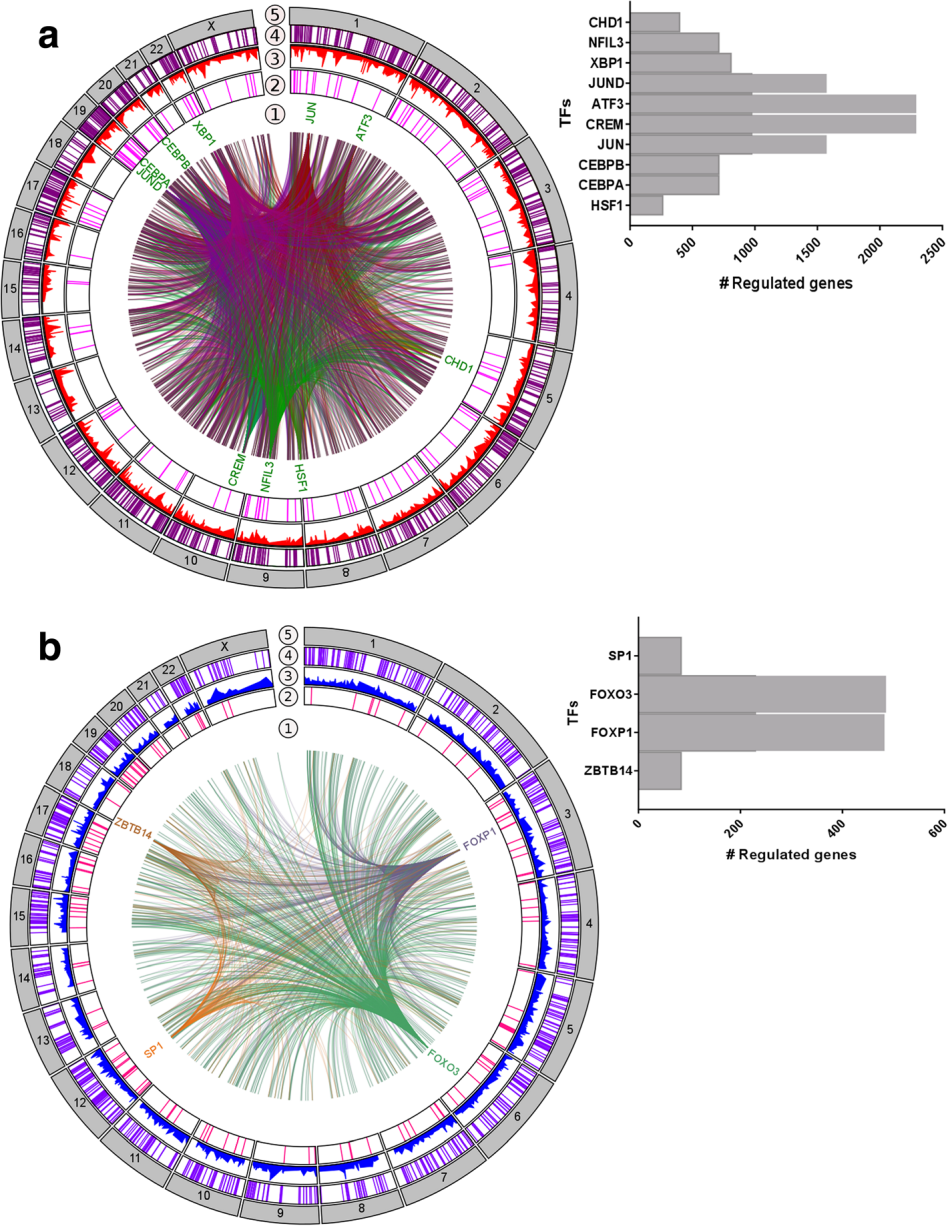
Regulatory network analysis reveals a set of immediate-early genes that are acutely responsive to NE stimulation. Integrated view of relationships between master (1) and local (2) regulators, their expression (3) and their predicted targets (4) mapped to the chromosomal context (5). Regulatory edges link the master regulators to their targets, which were colored as indicated in the figure. Up-regulated (**a**) and down-regulated (**b**) regulatory networks, based on acute responses to NE stimulation, revealed master regulators with different targets, as shown in histograms

Due to the fact that IEGs do not require de novo protein synthesis for their expression, that they acutely respond to NE stimulation, and that they are TFs [22], we assume that our MRs may be IEGs. Several MRs, which we identified in our dataset as up-regulated, are widely acknowledged as IEGs in general, like FOS, JUN, JUND, ATF3, CREM, CEBPA, and CEBPB (previous section and Fig. 3a), thus serving as validation of our approach. Several of these MRs are known to be involved in WAT conversion to the brite phenotype, such as CREM, CEBPA, and CEBPB [46, 47]. Moreover, in our TRN we also identified TFs which are not yet recognized as IEGs, but were acutely responsive to NE, such as the heat shock transcription factor 1 (HSF1) and the nuclear factor, interleukin 3-regulated (NFIL3). Interestingly, the highest ranked MR (NES = 5.5) was HSF1, a TF involved in the transcriptional activation of the heat shock response (HSR) [48] and known to regulate energy expenditure through activation of a PGC1α-dependent metabolic program [7]. Another highly ranked MR is NFIL3, a transcriptional regulator known for its role in circadian rhythm [49].

Moreover, the TRN of down-regulated genes identified four TFs as MRs, with ZBTB14 as the highest ranked (NES = 4.6), followed by FOXP1, SP1 and FOXO3 (Fig. 3b). Several pro-adipogenic factors, like PPARG, HOXC8, HOXB3, HOXA9, RARG and KLF5, are LRs downregulated upon NE treatment, suggesting a shutdown of the adipogenic program.

Thus, our results indicate that several TFs, including MRs and LRs, form a TRN that participates in the regulatory program of white adipocytes, with a shutdown of pro-adipogenic TFs and the initiation of pleiotropic genes capable of altering cell fate, including a number of factors known to be involved in the remodeling of white adipocytes. Moreover, our results pinpoint HSF1 and NFIL3 as promising novel IEGs acutely triggered by NE in primary human white adipocytes.

### Functional enrichment analysis elucidates a complex response to NE in metabolic and signaling pathways

**Fig. 4:**
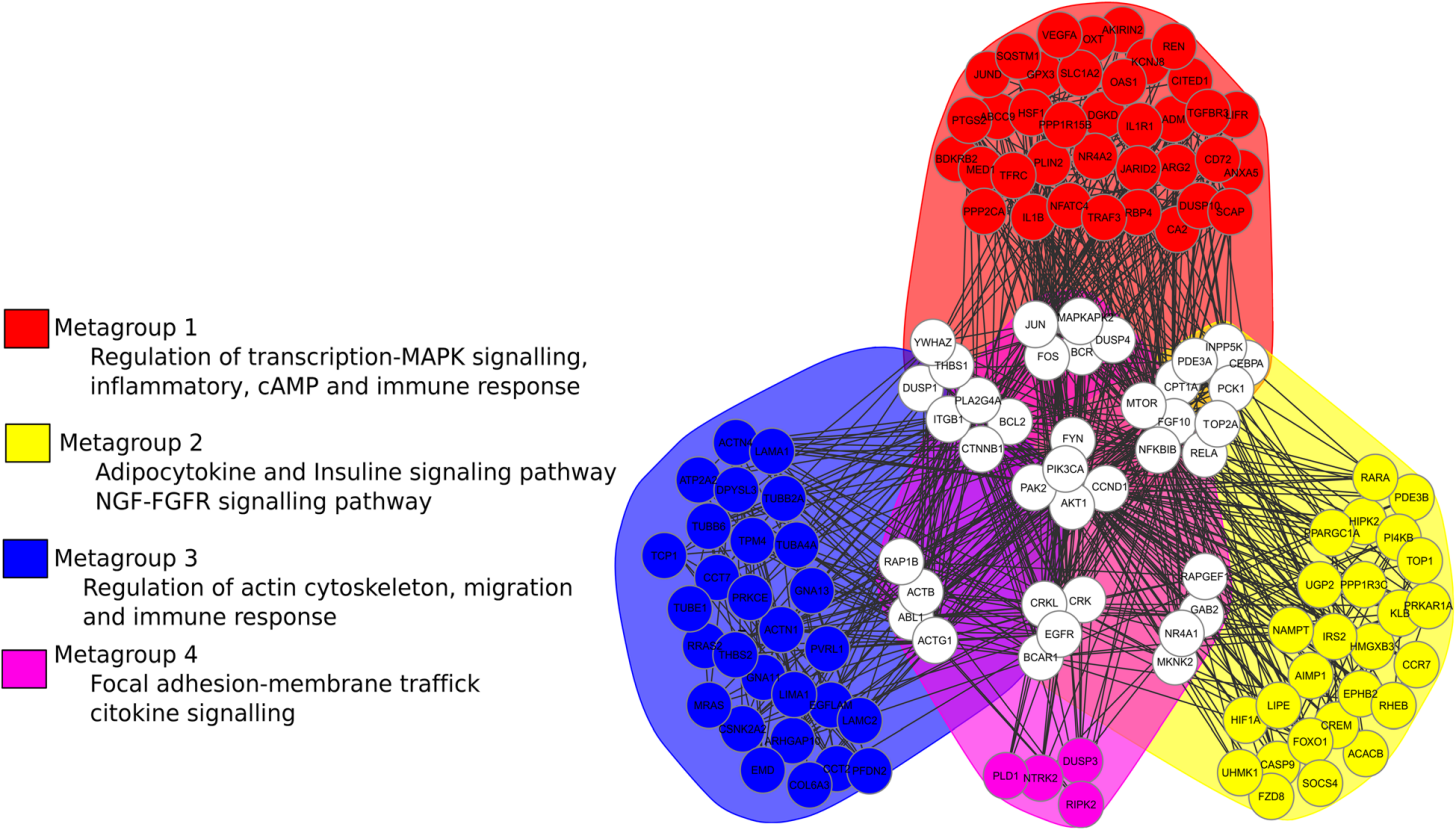
Functional enrichment analysis suggests a complex changes in metabolic and signaling pathways upon acute NE stimulation. Functional gene network analysis of up-regulated genes showing metagroup enrichment in distinct cellular functions as represented by colored nodes and clusters. White nodes represent genes that connect two or more functional modules. Metagroup compositions are depicted in Table 1

Our results revealed that NE stimulation up-regulated several IEGs that we also identified as MRs and LRs with pleiotropic function. Thus, we speculated whether they participate in the crosstalk between pathways and performed a functional gene enrichment analysis of the up-regulated DEGs using the R/Bioconductor package FGNet [50], reconstructing the functional gene network from up-regulated genes. FGNet which also organized the network into metagroups that condense multiple KEGG pathways, Reactome pathways, and GO terms (Fig. 4). The genes clustered in four metagroups. These metagroups contain genes classified according to their functions and seven clusters of common genes that are part of two or more metagroups. In each cluster, there are TFs, adaptor proteins, kinases as well DNA binding proteins (Table 1).

**Table 1:**
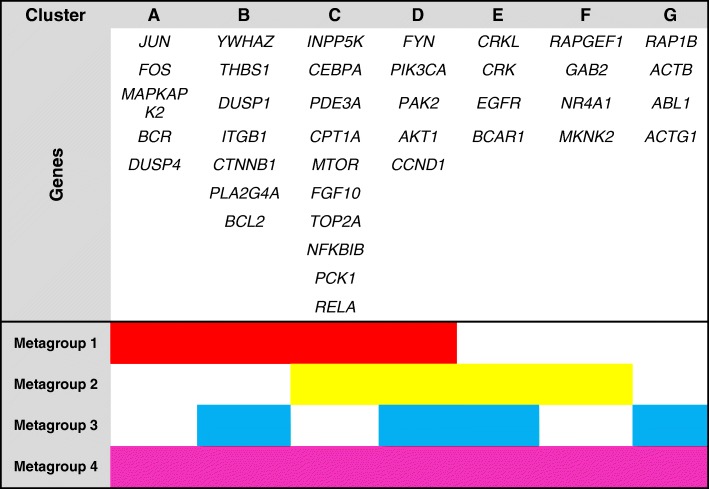
Functional module composition. Each metagroup represents cellular functions, each represented by a specific color and composed of several gene clusters. A total of four metagroups and seven clusters were identified

Because numerous genes tightly interconnect the four metagroups, our results suggest a crosstalk between pathways after NE stimulation. In conclusion, our approach provides hypotheses regarding the connections between functional metagroups. These connections can be used as the basis for investigating the mechanisms that link these functionally related genes. We expect that as more data become available, these networks will be further refined and expanded to provide a more in-depth insight into the regulatory network that drives biological function.

## Discussion

Despite the key role of NE in the sympathetic nervous system, acute transcriptional processes unleashed by NE in human models have been barely studied. In the present work, we investigated the acute response to NE stimulation in the transcriptional landscape of human primary white adipocytes with the aim to identify immediate-early genes and their downstream network, as well as their relationship to biological pathways that may ultimately explain phenotypical changes that have been described as response to NE in previous studies.

Primary adipose-derived stem cells from four donors have been studied. In order to diminish this limitation in available donor samples, hpASC from donors were randomized and treated as repetitions, to dilute the batch effect and the differences between individuals. Our approach allowed us to identify significant early responders to NE, with some of them being known responders that serve as positive controls, and with some of them identified in this study as novel responders to NE. However, because adipocytes from female individuals only were studied, conclusions on sex-dependent responses to NE are beyond the scope of this study. Despite these limitations, the results of our study suggest important changes on the cellular transcriptional network, and we identified known and novel IEGs and pathways to be involved in early NE response.

Another issue is the contamination of adipocyte populations with macrophages when adipocytes are directly isolated from tissue. In order to avoid this problem, we isolated primary adipose-derived stem cells (hpASCs) that were subsequently differentiated into white adipocytes to ensure a majority population of adipocytes. Moreover, after sequencing, we searched for the expression of several macrophage markers, but their expression levels were magnitudes lower than those of adipocyte markers (Additional file 2: Figure S1) so that we can assume that the contribution of macrophages to the expression signature in our dataset is negligible in comparison to adipocytes.

Over the last decades, several studies focused on lipid and glucose mobilization triggered by NE in WAT through the ADRβ/cAMP/PKA/PI3K axis [51], and it was assumed that these functions were performed mostly without the need for “de novo” transcription. However, our results show that several TFs are acutely transcribed upon NE treatment, together with more than 2,000 DEGs, indicating an acute shift in gene regulation and cell fate after NE stimulation.

Transcriptional regulation in adipocytes has been intensely studied in recent years on brite adipocytes [52, 53]. Several lines of evidence indicate in mouse [54, 55] and human [56] that chronic adrenergic stimulation or agonistic activity triggers conversion of white adipocytes to a brown-like phenotype with thermogenic activity due to high expression and functionality of UCP1 [57, 58]. Most adipocyte studies have analyzed adipocyte responses to stimulation by NE or agonistic activity [59] after 24 to 72 h in mouse or cell systems, and after weeks in humans [55, 56], but not after 3 h as we did. Therefore, it was surprising that we found some of the considered brite adipocyte marker genes, such as CITED1, HOXA9, DIO2 or PPARGC1A [60] already being up-regulated at such an early stage of NE stimulation. In line with that, transcription of several white adipocyte marker genes, such as PPARG, RUNX1T1 or HOXC8 [61, 62] were repressed, as well as genes involved in osteogenesis, such as SMAD3, 5 and 9 [63, 64] (Additional file 3: Table S4). These results suggest, provocatively, that NE-driven conversion starts already at an earlier stage than expected.

At pathway level, we found an up-regulation of growth factors such as NGF, PDGF, VEGF, WNT and circadian clock factors. NGF, PDGF and VEGF are well-known growth factors that share a common signaling cascade, the Akt/mTOR/MAPK axis [65]. Intriguingly, there are lines of evidence which indicate that these factors are critical for cell fate and tissue dynamics [66–68].

On the other side of the spectrum, NE stimulation led to a down-regulation of TP53, chromatin organization, and programmed cell death pathways. In principle, these findings are not surprising, because it is necessary to shut down the cell padlocks in order to initiate a change in the cellular program [69, 70] and to avoid the induction of cell death [71].

The fact that not a large number of DEGs in our study could be assigned to gene and pathway annotations, coincides with an increasing body of evidence suggesting that canonical pathways are incomplete and inaccurate models to study the complex interplay of signal transduction, transcriptional, post-transcriptional, metabolic and other regulatory events that determine cell behavior [72]. A possible and widely used solution to analyze information flow in systems biology is network construction, since networks act as a paradigm for data integration and analysis, providing a systems-level understanding of the mechanisms underlying cell biology and disease [73, 74]. The interaction network of DEGs upon NE stimulation pinpointed several highly connected nodes based on betweenness. We could identify genes with several functions: those codifying for signaling transduction proteins (MAPK14, SOS1, ABL1), structural proteins (ACTB), cell cycle (CDK1, BCL2), metabolic signaling proteins (PIK3CA, AKT, mTOR), post-translational modifiers (UBE2D1, YWAHZ), and specific TFs (NCOR2, RELA, SMAD3, FOXO1, JUN, FOS, YAP1). Nodes with high betweenness have been demonstrated to play a central role in biological networks [75–77], and are frequently recognized as pleiotropic or essential genes, and their differential expression can predict information flow [78].

Regulatory network analysis based on TF motif activity has been used before ranking and dissecting the influence of TFs in biological networks [79]. Just as in interaction networks, bottlenecks in regulatory networks are highly related to their role as essential proteins with the addition of having implicit flow, since their edges are directed. Our study identified several TFs that could play a role as master and local regulators in white adipocytes upon NE stimulation, many of them being well-known IEGs (for instance, FOS, JUN, JUND, ATF3 or CEBPA/B). FOS and JUN are responsive to adrenergic stimulation in rat cardiomyocytes [40] suggesting a conserved regulatory response to NE between rat and human, in cardiomyocytes and adipocytes. Nevertheless, the bulk of knowledge about IEGs and their regulation is based on studies of neurons and the nervous system [80–82]. In those models, the main functions described are proliferation, differentiation, survival [83], and stem cells fate [84].

An unexpected result of our TRN analysis was the identification of HSF1 and NFIL3 as novel IEGs and MRs in white adipocytes. HSF1 is a TF that plays a central role in the transcriptional activation of the heat shock response (HSR), leading to the expression of heat shock proteins. Notably, HSF1 has previously been identified as an important factor in white to brite conversion after seven days of stimulation with celastrol [7], and its ablation impairs energy metabolism [85, 86]. Moreover, it has been described as a central regulator of cellular bioenergetics and protein homeostasis in liver [87]. Thus, our work pinpoints HSF1 as a novel IEG candidate that could regulate adipocyte energetics upon NE stimulation at very early stages. NFIL3 is a transcriptional regulator, mainly known for being involved in circadian regulation [88] and shutting down apoptotic signals promoting survival and regeneration [89]. While it is a known responder to adrenergic stress in fibroblasts, it has also been demonstrated to be induced by activation of the α1-adrenergic receptor and does not involve the cAMP pathway [90, 91]. Taken together, our results suggest that the acute response to NE stimulation is coordinated by several adrenergic receptors and pathways.

We performed a functional enrichment network analysis in order to identify specific functional modules and a crosstalk between identified pathways. The identification of genes that work as “bridges” or linkers between the four identified metagroups reveal not only TFs but also other molecules, such as kinases, receptors and even structural genes that can be critical regulators of cell processes. Although it is true that we do not have a quantitative way to support whether these metagroups have an impact to drive the phenotype, biological evidence shows that several genes participating in crosstalk make sense in cellular reprogramming and cell fate, as BCR on B cells [92], CCND1 on epidermal cells [93] or mTOR on pancreatic cells [94]. High redundancy in the signals across several functions, like immune response or signaling events was observed, where diverse clusters of transcriptional and post-transcriptional regulators seem to control a robust network. Our results not only allow the inference of transcriptional regulation, but also of post-transcriptional effectors, such as the YWHAZ, a gene encoding for the 14–3-3ζ protein, a signal adapter capable of functioning as a kinase and allowing the flow of information in signaling cascades on effectors like PI3K [95], mTOR or AKT [96].

## Conclusions

We identified a broad transcriptional response of primary human white adipocytes to acute NE stimulation. With our reconstruction of functional, interaction, and transcriptional networks, we identified novel NE-responsive bottleneck genes with high betweenness, novel NE-responsive immediate-early gene candidates, and a complex response to NE in metabolic and signaling pathways. Our study provides a basis for hypothesis-driven studies on how the activation of IEGs orchestrates the shift in the transcriptional program after acute NE stimulation, and the amplitude of its effects in the cellular network of adipocytes. In future studies, the use of a cohort can help to infer possible sex differences and stratification to NE response between populations. Studies with human samples offer particular challenges; nevertheless, functional genomic assays could lead to an impact on a variety of practical applications such as the identification of novel candidate genes capable of modulating cell fate in response to extrinsic signals, the discovery and exploration of non-canonical pathways, and a deeper insight into crosstalks between metabolic and transcriptional networks

## Methods

### Isolation and culture of hpASCs

Human primary adipose-derived stem cells (hpASCs) were isolated from subcutaneous lipoaspirates from healthy female donors (*n* = 4) as previously described [97]. Cells at P0 were thawed, cultivated in EGM-2 Medium (Lonza), and used after 1–3 passages. For adipocyte differentiation, cells were seeded in 6-well plates (85000 cells / well) in EGM-2 Medium. Cells reached 100% confluence after 3 days, when the medium was replaced with fresh EGM-2 Medium. 2 days later (= day 0), adipocyte differentiation was induced by changing the medium to adipocyte differentiation (AD) medium (DMEM/Ham’s F12 (50:50), 5 mM HEPES, 2 mM L-glutamine, 100 μg/ml normocin, 860 nM insulin, 10 μg/ml apo-transferrin, 100 nM rosiglitazone, 0.2 nM triiodothyronin) supplemented with 100 μM 3-isobutyl-1-methylxanthine (IBMX), and 1 μM dexamethasone (Dex). Medium was replaced at days 2 and 5 with AD medium. At day 7, another medium change was performed using AD medium without insulin. Adipogenesis was monitored and confirmed based on the appearance of lipid droplets by light microscopy.

### Acute norepinephrine (NE) stimulation

Based on literature, we chose the most widely used NE concentration and the time point which demonstrated an acute reaction to NE [98]. At day 9 of differentiation, norepinephrine (NE) stimulation was performed by changing the medium to AD medium without insulin, but supplemented with 25 μg/mL ascorbate and either 1 μM norepinephrine (NE; dissolved in 10 mM HCl) or vehicle (VE, 10 mM HCl).

### RNA extraction and sequencing

Cells were harvested with TRIzol reagent (Invitrogen) 3 h after stimulation with NE, and RNA isolation was performed according to the manufacturer’s protocol. Quality of RNA was assayed by an RNA Nano chip using the BioAnalyzer 2100 (Agilent); all samples had RIN values ≥8.5. Four μg total RNA per sample were used for the TruSeq Stranded mRNA LT Sample Prep Kit (Illumina) to generate cDNA libraries according to the manufacturer’s protocol. Single read sequencing was carried out using Illumina/Solexa HiSeq 2000. High-throughput sequencing was conducted by the Biomedical Sequencing Facility (BSF) at CeMM in Vienna.

### RNA-seq alignment and differential expression analysis

Raw RNA sequencing reads were aligned against the human hg38 genome using STAR aligner with default parameters [99]. The mapped reads were assigned to genes using featureCount from the bioconductor package Rsubread [100]. All annotated genes were quantified across each condition, corresponding to the University of California at Santa Cruz (UCSC) GRCh38.84 annotation. Normalization and differential expression analysis were performed using the R/Bioconductor package DESeq2 [101]. The effect size / differential expression threshold was situated in an absolute log_2_ fold change of at least 0.19 (1.17 FC) and an adjusted *p*-value (padj) < 0.01.

### Principal component analysis (PCA) and hierarchical clustering

Principal component analysis (PCA) was performed using the procedure implemented in the R function prcomp in the normalized RNA-seq counts. Unsupervised hierarchical clustering of the RNA-seq data was performed with a centered Pearson correlation coefficient algorithm and a complete linkage method using the R function Heatmap.

### Pathway analysis

Pathway enrichment analysis was performed using the R/Bioconductor package ReactomePA, version 1.22.0 [102]. *P*-values were adjusted for multiple comparisons using the Benjamini-Hochberg procedure.

### Network reconstruction

Network reconstruction was performed in Cytoscape with the BisoGenet plugin [36, 103], using DE genes as bait nodes. Edges from experimentally validated interactions were added using the following parameters: Organism > *Homo sapiens*, gene identifiers only; Data Settings > protein-protein interactions; DIP, BIOGRID, HPRD, INTACT, MINT and BIND databases and all experimental methods; Protein-DNA interaction from experimentally validated interactions; BIND and ENCODE databases, all experimental methods; Method > Criteria to build the network, connecting input nodes with the “By adding edges” option, and as Output > Genes.

### Betweenness analysis

The Cytoscape plugin Cyto-Hubba [37] was used to analyze the network topologies and to calculate betweenness centrality. DEGs receiving the highest scores in betweenness were selected as the highest-ranked genes in the network and visualized in their network context using atlas force layout.

### Transcription factor and regulatory network analysis

We performed a gene based motif enrichment analysis of our network by using the Cytoscape iRegulon plugin [45]. The following parameters were used: Species> *Homo sapiens*; Search space> gene-based; Motif collection> 10 k; Track collection> 1120 ChIP-Seq tracks; Putative regulatory region> 20 kb centered around TSS; Motif ranking database> 20 kb centered around TSS (10 species); Track ranking database> 20 kb centered around TSS (ChIP-Seq derived); Region-based parameters>default; Recovery parameters: Enrichment score threshold> 3.0; ROC threshold for AUC calculation > 0.03; Rank threshold > 5000; Transcription factor prediction: Minimum identity between orthologous genes > 0.0 and Maximum FDR on motif similarity > 0.001. Circos plots of predicted transcriptional networks were created using Circa software (OMGGenomics, 2017).

### Functional network construction

We constructed a network derived from a Functional Enrichment Analysis (FEA) using DAVID Functional Annotation Clustering (DAVID-FAC), clustering genes in groups of highly related terms [104]. Visualization of the functional gene network and metagroup inference were performed by the R/Bioconductor package FGNet [50].

### Statistical analysis

Individual changes in immediate-early gene expression after NE stimulation were determined using RNA-seq read counts normalized by counts per million (CPM). Comparisons among groups were performed using a Kruskall-Wallis test followed by a post-hoc Dunn’s multiple comparison test. All the statistical analyses and graphics were performed using R, version 3.3.3 (2017-03-06).

## Additional files

Additional file 1: **Table S1.** Genes with highest betweenness. **Table S2.** Local and Master regulators up-regulated. **Table S3.** Local and Master regulators down-regulated (XLSX 23 kb).

Additional file 2: **Figure S1.** Adipocyte and macrophage marker gene expression in white adipocytes in the unstimulated and 3 h NE-stimulated state. Y-axis indicates the transcript abundance in Transcript Per Million (TPM) (PDF 41 kb).

Additional file 3: **Table S4.** Counts per Gene per Million Reads Mapped. (XLSX 2751 kb).

## Abbreviations

cAMP: Cyclic adenosine monophosphate
CPM: Counts per million
DEG: Differentially expressed genes
hpASCs: Human primary adipose-derived stem cells
IEGs: Immediate-early genes
log2FC: Logarithm 2-fold change
LRs: Local regulators
MRs: Master regulators
NE: Norepinephrine
NES: Normalized enrichment score
NGF: Nerve growth factor
PCA: Principal component analysis
PDGF: Platelet-derived growth factor
PKA: Protein kinase
PPI: Protein-protein interaction
TFs: Transcription factors
TRN: Transcriptional regulatory network
WAT: White adipose tissue
