# Supplementary material for: Norepinephrine triggers an immediate-early regulatory network response in primary human white adipocytes
Source: BMC Genomics. 2018 Nov 3;19:794. doi: 10.1186/s12864-018-5173-0 (PMC6215669; doi:10.1186/s12864-018-5173-0)
Supplement: Supplementary file 2 — Figure S1. Adipocyte and macrophage marker gene expression in white adipocytes in the unstimulated and 3 h NE-stimulated state. Y-axis indicates the transcript abundance in Transcript Per Million (TPM) (PDF 41 kb). [file 12864_2018_5173_MOESM2_ESM.pdf]

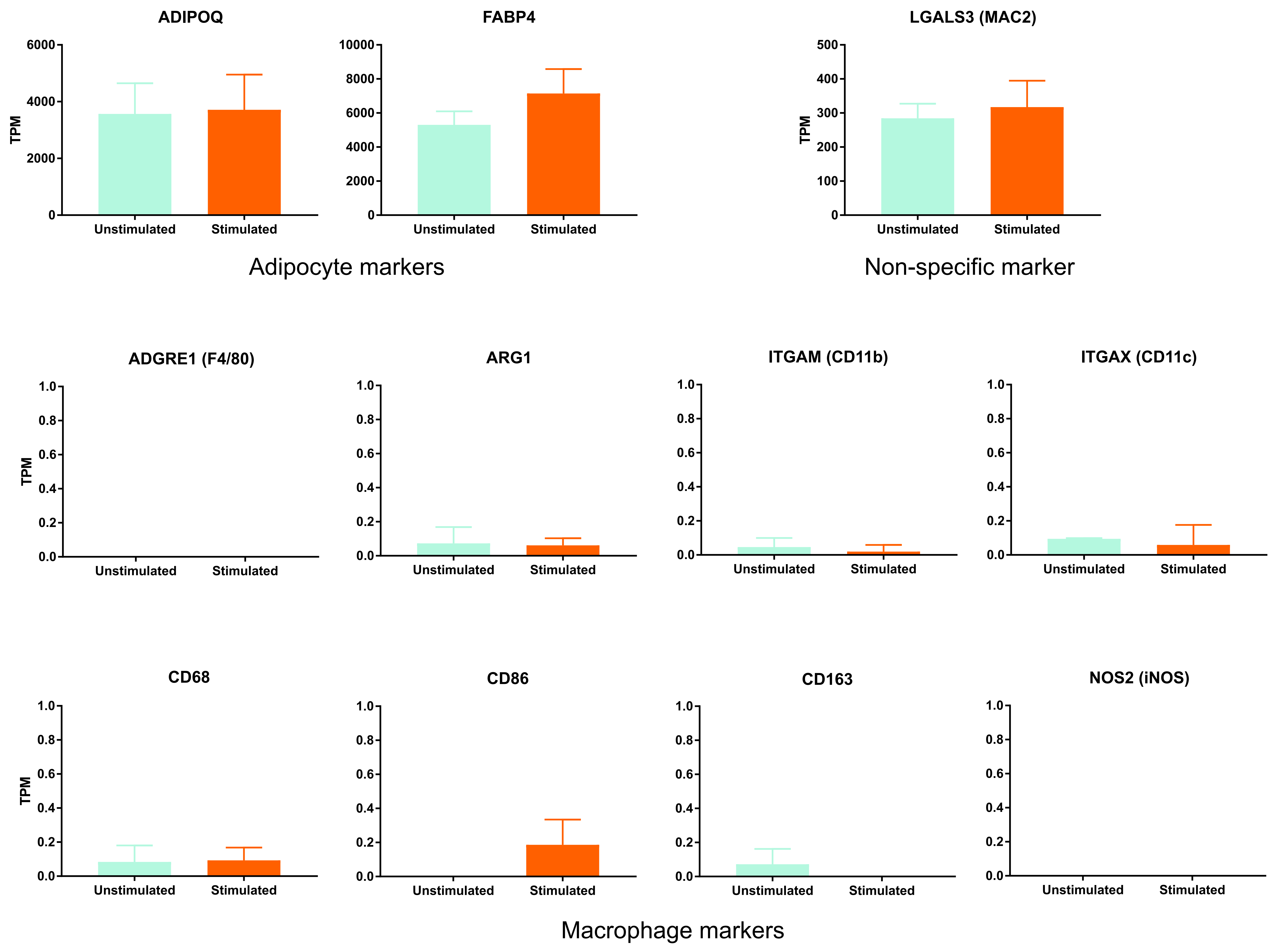

ADIPOQ
FABP4
LGALS3 (MAC2)
TPM
Unstimulated
Stimulated
Adipocyte markers
Non-specific marker
ADGRE1 (F4/80)
ARG1
ITGAM (CD11b)
ITGAX (CD11c)
CD68
CD86
CD163
NOS2 (iNOS)
Macrophage markers
